# Pretreatment myopia progression is associated with the response to 0.01% atropine: a *post-hoc* secondary analysis of a randomized controlled trial

**DOI:** 10.3389/fphar.2026.1871905

**Published:** 2026-08-26

**Authors:** Shi-Fei Wei, Wei-Ling Bai, Yun-Yun Sun, Wen-Zai An, Xin-Tong Liang, Jia-He Gan, Zhi-Ning Cai, Qian-Qian Song, Shi-Ming Li, Ning-Li Wang

**Affiliations:** 1 Beijing Institute of Ophthalmology, Beijing Tongren Eye Center, Beijing Key Laboratory of Ophthalmology and Visual Science, Beijing Tongren Hospital, Capital Medical University, Beijing, China; 2 Department of Ophthalmology, Beijing Shijitan Hospital, Capital Medical University, Beijing, China; 3 Department of Ophthalmology, Peking University First Hospital, Beijing, China; 4 Department of Ophthalmology, Shanghai East Hospital, School of Medicine, Tongji University, Shanghai, China

**Keywords:** 0.01% atropine eye drops, children, myopia progression, personalized myopia management, *post hoc* analysis

## Abstract

**Purpose:**

To determine which subgroup of children would best benefit from 0.01% atropine eyedrop.

**Methods:**

This *post hoc* secondary analysis assessed 68 subjects. Placebo for the first year and 0.01% atropine eye drops for the second year. According to the annual spherical equivalent progression (SEP) during the first year, subjects were subdivided into three groups: slow, moderate and fast (annual SEP > −0.50 D, −1.00 D < annual SEP ≤ −0.50 D, annual SEP ≤ −1.00 D, respectively).

**Results:**

After 1 year of 0.01% atropine treatment, SEP was −0.37 ± 0.23 D, −0.57 ± 0.38 D, and −0.68 ± 0.49 D in the slow, moderate, and fast progression groups (P = 0.17). The group with faster SEP during the first year still showed faster SEP after treatment. The reduction of SEP (SEP of the second year minus SEP of the first year) in the moderate group was more than that in the slow group (0.22 ± 0.37 D vs. −0.10 ± 0.21 D, P = 0.007); the fast group was more than that in the slow group (0.57 ± 0.55 D vs. −0.10 ± 0.21 D, P < 0.001) and the moderate group (0.57 ± 0.55 D vs. 0.22 ± 0.37 D, P = 0.02). Faster SEP before treatment is related to the reduction efficiency (β = −0.5, P = 0.01).

**Conclusion:**

Faster SEP before treatment is associated with a better treatment response to 0.01% atropine eye drops, with greater reduction in SEP after treatment. However, no significant association was found between baseline progression and axial elongation changes. Children with slow SEP cannot get significant myopia control benefit from 1-year 0.01% atropine treatment.

**Clinical Trial Registration:**

clinicaltrials.gov, identifier ChiCTR-IOR-17013898.

## Highlights

- Myopia progression rate before treatment is an important but understudied factor affecting the efficacy of 0.01% atropine eye drops in children.
- This *post hoc* secondary analysis aimed to determine whether pretreatment myopia progression is associated with a better treatment response the response to 0.01% atropine eye drops.
- Faster pretreatment myopia progression was associated with significantly greater reduction in myopia progression after 0.01% atropine treatment. While, no significant association was found between baseline progression and axial elongation changes.
- Children with slow baseline myopia progression derived no significant myopia control benefit from 1-year 0.01% atropine treatment.
- These findings support personalized myopia management by stratifying children according to pretreatment progression speed.

## 1 Introduction

Myopia is a sight-threatening public health problem, and the increasing prevalence of myopia has brought significant economic and social burdens (Holden et al., 2016; Baird et al., 2020; Wei et al., 2018). Currently, clinical guidelines for myopia control include orthokeratology lenses, contact lenses with peripheral defocus design, maximizing time spent outdoors, and low-concentration atropine eye drops (Chen et al., 2022; Fricke et al., 2022; Gispets et al., 2022; Jonas et al., 2021; Li et al., 2015; Wei et al., 2020; Li et al., 2022; Li Q. et al., 2021). The use of atropine to prevent myopic progression is supported by Level I evidence, it has been proven to be one of the most effective treatments for myopia (Pineles et al., 2017; Joint World Health Organization-Brien Holden Vision Institute Global Scientific Meeting on Myopia et al., 2015). In the Atropine for the Treatment of Myopia (ATOM) study, 0.01% atropine eye drops were more effective in slowing myopia progression with less visual side effects compared with higher doses of atropine (Chia et al., 2016). 0.01% atropine eye drops reduced axial elongation after a 3-year treatment in the Childhood Atropine for Myopia Progression (CHAMP) phase 3 (Repka et al., 2023).

Clinically, studies have reported children who showed progressive myopia despite treatment with 0.01% atropine eye drops (SEP < −0.5 D) (Chia et al., 2016; Pérez-Flores et al., 2021; Jeon et al., 2021). Wei and associates showed that 13.2% in the 0.01% atropine group progressed by at least 1.00 D after 1 year of treatment (Wei et al., 2020). In phase 1 of the ATOM, 13.9% of atropine-treated eyes had a SEP ≤ −0.5 D (Chua et al., 2006). 27.8% of subjects in the 0.01% atropine group progressed by ≤ −1.00 D in the Low-Concentration Atropine for Myopia Progression (LAMP) Study (Yam et al., 2019). In a multicenter study conducted among Spanish children, 5.4% patients underwent a progression in the SE ≤ −1.00 D over a 1-year period (Pérez-Flores et al., 2021).

In clinical practice, which specific subgroup of children would best benefit from low-dose atropine treatment (e.g., age, level of myopia, rate of progression, pupil diameter, and family risk factors) remains uncertain (Chia et al., 2016; Bai et al., 2023). At present, there is a need to establish the clinical management guideline for 0.01% atropine eye drops based on the expected effect of treatment. Children with younger age, a higher degree of myopia at baseline, starting spectacle wear at a younger age, and a history of parental myopia was associated with a poorer response to atropine treatment (Chia et al., 2016; Wu et al., 2011; Loh et al., 2015). Using an online survey to map the practice patterns for controlling myopia progression, Leshno and colleagues found that the most common indication for treatment was the SEP (diopter/year) (Leshno et al., 2020). The Singapore Cohort Study of the Risk Factors for Myopia reported that compared to baseline spherical equivalent and age of myopia onset, 1-year myopia progression had the highest area under the curve for predicting following 2-year myopia progression (Matsumura et al., 2020). While, in the predictive models derived from the Collaborative Longitudinal Evaluation of Ethnicity and Refractive Error (CLEERE) Study data, the prior year’s myopia progression was weakly correlated with the next year’s progression (Mutti et al., 2022). As reported in a previous review, there is a lack of evidence to suggest that whether faster progressors derive greater benefit from various myopia control strategies (Zhang et al., 2025). Our study aimed to investigate whether the efficacy of 0.01% atropine eye drops is associated with baseline progression rates.

## 2 Methods

### 2.1 Study design

This *post hoc* secondary analysis was based on a randomized, double-masked, placebo-controlled, cross-over trial comprised of 2 phases in mainland China. The study was conducted between 2018 and 2020, the detailed design and methods have been described previously (Wei et al., 2020; Wei et al., 2022). In brief, children aged 6 to 12 have a spherical equivalent (SE) refraction range of −1.00 D to −6.00 D in both eyes, astigmatism of less than 1.50 D in both eyes, and intraocular pressure of less than 21 mmHg were enrolled in this study. In phase 1 (the first year), 220 subjects were randomized to receive either placebo or 0.01% atropine eye drops at bedtime every night in both eyes for 1 year. In phase 2 (the second year), the placebo group was crossed over to the 0.01% atropine group (referred to as the “placebo-atropine group”), and the 0.01% atropine group was crossed over to the placebo group (referred to as the “atropine-placebo group”) for 1 year. All eye drops were prepared in mono-dose preparation by Shenyang Xingqi Pharmaceutical Co, Ltd (Shenyang, China).

### 2.2 Participants

Participants in this study were drawn from the placebo-atropine group during these 2 years. The first-year spherical equivalent progression (year-end SE minus year-start SE, SEP) was calculated for each subject. According to their first-year SEP, subjects were subdivided into three groups: slow progression (defined as SEP > −0.50 diopters (D)), moderate progression (−1.00 D < SEP ≤ −0.50 D) and fast progression (SEP ≤ −1.00 D). Of 110 participants, 27 of them did not attend the follow-up in Phase 1, and 15 of them were lost to follow-up in Phase 2. The final analyzed cohort comprised 68 children (21 slow progression, 27 moderate progression, 20 fast progression) who completed the 2-year follow-up in the placebo-atropine group. We analysed only the placebo-atropine arm because it offered an untreated first year to determine natural progression. The atropine-placebo arm was excluded as its first-year progression was already confounded by active treatment. A CONSORT-style participant flow diagram was supplemented as Supplementary Figure S1 to illustrate participant allocation and exclusion.

The study adhered to the tenets of the Declaration of Helsinki and was approved by the Ethics Committee of Beijing Tongren Hospital. All participants provided written informed consent after agreeing to enrollment, which included permission for future secondary analyses of the research data. The trial was registered with the Chinese Clinical Trial Registry (http://www.chictr.org.cn/index.aspx). The registration number is ChiCTR-IOR-17013898 (Wei et al., 2020). The original RCT is the fundamental data source of this *post hoc* secondary analysis, so we still retain the trial registration number as required by academic norms.

### 2.3 Outcome measurements

During these 2 years, we followed the subjects every 6 months. Axial length (AL) was measured at each visit on a Lenstar LS900 (Haag-Streit Koeniz, Switzerland). The subjects were asked to blink completely to ensure an optically smooth tear film over the cornea then fixated on the target, the device was focused based on the image of the eye on the monitor. Measurements contaminated by blinking or unstable fixation were excluded and the average of five measurements was recorded. We performed the cycloplegic procedure with three drops of 1% cyclopentolate at a 5-min interval. Then, an autorefractor (HRK7000 A; Huvitz, Gunpo, South Korea) was used to measure the SE 3 times consecutively, with average data used for analysis. All three readings should be at most 0.25 D apart in both the spherical and cylinder components. The measurement of SE was performed 30 min after installing the third drop of 1% cyclopentolate. If the pupillary light reflex was still present or the pupil size was less than 6.0 mm, the fourth drop of 1% cyclopentolate would be given 30 min after the third drop. We tested the pupillary light reflex and the pupil size again after 15 min. At the baseline, parents were asked to answer an interviewer-administered questionnaire to collect their children’s information on the age of myopia onset (years), time near work (hr/day), and outdoor activities (hr/day) after school hours (Li et al., 2013).

### 2.4 Statistical analysis

Mean values were derived from the right eye. The SE was calculated as the dioptric powers of the sphere and half of the cylinder (sphere + 0.5 × cylinder). Negative values of cycloplegic SE change indicate worsening of myopia progression. Statistical analyses were performed using commercial software (SPSS version 25.0; SPSS, Inc., Chicago, IL, United States). Mean values for ocular parameters were calculated from the right eyes. Categorical data were represented as counts (frequencies). Mean and standard deviation values were used to describe continuous variables.

Continuous variables were analyzed using appropriate statistical methods based on data distribution and assumptions. Age comparisons among groups were performed using one-way ANOVA. For other continuous variables, analysis of covariance (ANCOVA) was used with age as a covariate. When ANCOVA assumptions (homogeneity of variance, linearity, or normality of residuals) were violated, Generalized Linear Models (GLM) were employed as an alternative. The Chi-square test was used to assess the difference in gender among the three groups. The multivariable regression model investigated whether SEP before treatment contributed to myopia progression during 0.01% atropine treatment. Baseline cycloplegic spherical equivalent was incorporated as a covariate in the multivariable regression to adjust for the confounding effect of initial refractive status since advanced myopia naturally has less potential to progress further. Given the collinearity between refractive error progression and axial length elongation, which could confound multivariate regression analyses, axial elongation before treatment was not included as a potential explanatory variable in the analysis. Supplementary linear regression was conducted with mean-centred age and baseline myopia progression to construct an interaction term for testing effect modification. A two-sided P < 0.05 was considered statistically significant.

## 3 Results

Of the 110 participants initially assigned to the placebo-atropine group, 83 completed the first-year follow-up, and 68 completed the entire 2-year study period. The final analysed cohort comprised 21 slow, 27 moderate, and 20 fast progressors. No significant differences were found in gender, baseline spherical equivalent, baseline axial length, baseline intraocular pressure, baseline time near work and outdoors activities, between the follow-up subjects and dropout subjects (P > 0.05), except for age (10.12 ± 1.51 years vs. 9.23 ± 1.52 years, P < 0.05) and age at myopia onset (8.41 ± 1.50 years vs. 7.5 ± 1.79 years, P < 0.05, Supplementary Table S1). The three groups had comparable baseline spherical equivalent, axial length, intraocular pressure and other ocular parameters, with only significant age differences observed between fast progression group (9.44 ± 1.75) and the slow progression group (10.85 ± 1.19 years, P = 0.02) (Table 1).

**TABLE 1:** Baseline characteristics of each progression group.

​	Group, mean (SD)			
	Slow myopia progression (n = 21)	Moderate myopia progression (n = 27)	Fast myopia progression (n = 20)	P value
Age (years)	10.85 (1.19)	10.06 (1.33)	9.44 (1.75)	0.02*
Sex (male, %)	13, 61.9%	15, 55.6%	6, 30.0%	0.11
Intraocular pressure (mmHg)	16.40 (1.94)	16.29 (2.71)	16.05 (2.93)	0.68
Cycloplegic spherical equivalent (D)	−3.05 (1.54)	−2.51 (1.40)	−2.48 (1.32)	0.63
Axial length (mm)	24.95 (1.16)	24.78 (0.99)	24.49 (0.77)	0.66
Age at myopia onset (years)	8.40 (1.17)	8.64 (1.92)	7.70 (1.64)	0.44
Near work (hours per day)	4.05 (1.17)	3.20 (1.12)	3.40 (1.82)	0.81
Time outdoors (hours per day)	1.41 (0.57)	1.49 (0.65)	1.54 (0.73)	0.66

D, diopter; SD, standard deviation.

* The *post hoc* test showed that the age of the fast progression group (9.44 ± 1.75) is younger than the slow progression group (10.85 ± 1.19) (P = 0.02).

There were significant differences in the myopia progression pairwise comparison among the three groups in the first year (−0.27 D ± 0.17 years of the slow progression group, −0.79 D ± 0.12 years of the moderate progression group, and −1.26 D ± 0.26 years of the fast progression group, Table 2). Axial elongation was significantly greater in moderate progression group (0.43 ± 0.11 mm) and the fast progression group (0.50 ± 0.14 mm) than in slow progression group (0.22 ± 0.11 mm). The AL changes were not significantly different between moderate (0.43 ± 0.11 mm) and fast (0.50 ± 0.14 mm) progression groups (Table 2). After 1-year treatment of 0.01% atropine eye drops, no significant between-group differences were observed in either myopia progression or axial elongation among the stratified progression groups, as detailed in Table 2.

**TABLE 2:** Annual myopia progression and axial elongation for each progression group before and after treatment.

​	Slow myopia progression	Moderate myopia progression	Fast myopia progression	P values^†^ (2 vs. 1, 3 vs. 1, 3 vs. 2)
Myopia progression, D, mean (SD)				
Before treatment	−0.27 (0.17)	−0.79 (0.12)	−1.24 (0.25)	<0.001*, <0.001*, 0.001*
After treatment	−0.37 (0.23)	−0.57 (0.38)	−0.68 (0.49)	0.16, 0.16, 0.88
Axial elongation, mm, mean (SD)				
Before treatment	0.22 (0.11)	0.43 (0.11)	0.50 (0.14)	<0.001*, <0.001*, 0.32
After treatment	0.29 (0.17)	0.35 (0.18)	0.41 (0.18)	1.00, 0.80, 1.00

D, diopters; SD, standard deviation.

* Significance was set at 0.05.

^†^ Bonferroni correction was applied for the pairwise comparisons.

Supplementary multivariable linear regression analyses were performed to test whether age modifies the association between pretreatment myopia progression and treatment response. Baseline SEP and age were mean-centred prior to constructing the age-by-baseline-progression interaction term, with all predefined confounders (sex, age at myopia onset, near work, outdoor activity, baseline spherical equivalent) fully adjusted in the models. For the primary outcome of SEP reduction, the interaction term did not reach statistical significance (β = 0.04, 95% CI: −0.18 to 0.22, P = 0.83). No significant age-by-baseline-progression interaction was observed for axial elongation reduction either (β = −0.08, 95% CI: −0.13 to 0.09, P = 0.72). These supplementary findings indicated no detectable modifying effect of age on the predictive value of pretreatment myopia progression for 0.01% atropine efficacy. Given the limited sample size, these null results should be interpreted cautiously and do not rule out weak age-related effect modification.

**FIGURE 1:**
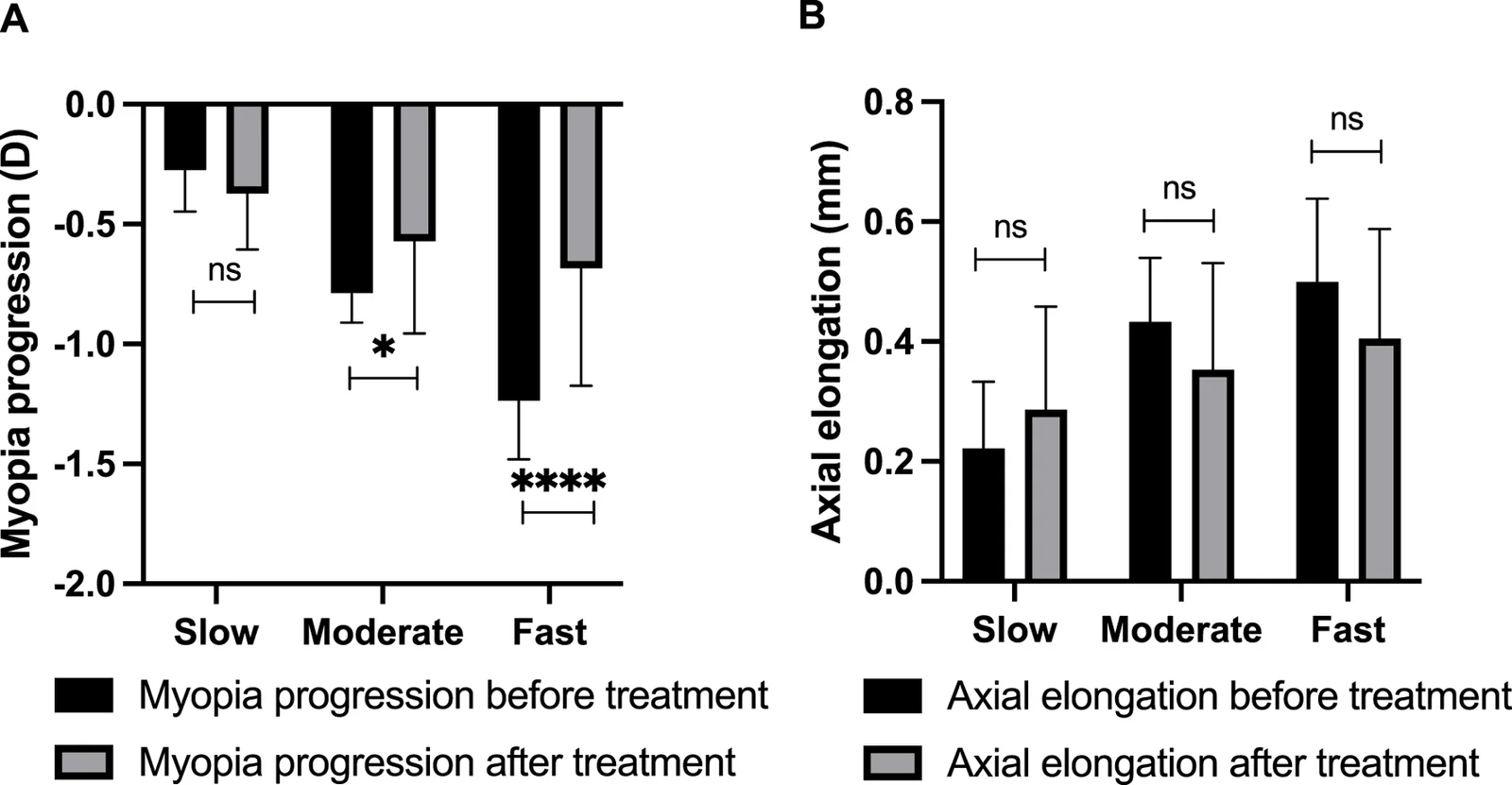
Comparison of myopia progression of spherical equivalent **(A)** and axial elongation **(B)** before and after the 0.01% atropine treatment among slow progression, moderate progression and fast progression group. Slow progression group (n = 21), moderate progression group (n = 27), fast progression group (n = 20). Error bars represent one standard deviation of the mean. *P < 0.05, ****P < 0.0001.

We compared the three groups’ SEP and axial elongation before and after treatment (Figure 1). There was no significant difference in myopia progression (−0.27 ± 0.17 D vs. −0.37 ± 0.23 D, P = 0.69) or axial elongation (0.22 ± 0.11 mm vs. 0.29 ± 0.17 mm, P = 0.26) before and after treatment of the slow progression group. The progression of myopia in the moderate progression group was significantly reduced after treatment (−0.79 ± 0.12 D vs. −0.57 ± 0.38 D, P = 0.04), there was a slight difference in the axial elongation before and after treatment (0.43 ± 0.11 mm vs. 0.35 ± 0.18 mm, P = 0.09). The progression of myopia in the fast progression group was significantly reduced after treatment (−1.24 ± 0.25 D vs. −0.68 ± 0.49 D, P < 0.001), there was no significant difference in the axial elongation before and after treatment (0.50 mm ± 0.14 mm vs. 0.41 mm ± 0.18 mm, P = 0.09).

The reduction (SEP of the second year minus SEP of the first year) in the moderate progression group was larger than that in the slow progression group (0.22 ± 0.37 D vs. −0.10 ± 0.21 D, P = 0.01). The reduction in the fast progression group was larger than that in the slow progression group (0.57 ± 0.55 D vs. −0.10 ± 0.21 D, P < 0.001) and the moderate progression group (0.57 ± 0.55 D vs. 0.22 ± 0.37 D, P = 0.02). Compared with the slow progression group, moderate progression group (−0.07 ± 0.17 mm vs. 0.07 ± 0.20 mm, P = 0.2) and fast progression group (−0.09 ± 0.16 mm vs. 0.07 ± 0.20 mm, P = 0.13) showed less axial elongation after treatment. The moderate and fast progression groups exhibited similarly less axial elongation (−0.09 ± 0.16 mm vs. −0.07 ± 0.17 mm, P = 1.00, Table 3).

**TABLE 3:** Reduction of myopia progression and axial elongation after one-year treatment.

​	Slow myopia progression	Moderate myopia progression	Fast myopia progression	P values^†^ (2 vs. 1, 3 vs. 1, 3 vs. 2)
Reduction of myopia progression after treatment	−0.10 (0.21)	0.22 (0.37)	0.57 (0.55)	0.007*, <0.001*, 0.02*
Reduction of axial elongation after treatment	0.07 (0.20)	−0.07 (0.17)	−0.09 (0.16)	0.20, 0.13, 1.00

D, diopters; SD, standard deviation. Positive values indicate a decrease in myopia progression, while negative values indicate a decrease in axial elongation. Data are presented as mean (standard deviation, SD).

* Significance was set at 0.05.

^†^ Bonferroni correction was applied for the pairwise comparisons.

Multivariable regression analyses assessed the factors associated with the effect of 0.01% atropine eye drops in terms of reducing myopia progression and axial elongation (Table 4). After adjusting for age, sex, age at myopia onset, near work and outdoor activity duration, the SEP before treatment remained an independent predictor of treatment response (β = −0.50, P = 0.01). Whereas no significant association was found for the reduction in axial elongation. It was shown that the faster the myopia progression before treatment, the more significant the reduction effect of the myopia progression brought by 0.01% atropine eye drops. Other variables did not show a significant effect on the reduction of myopia progression.

**TABLE 4:** Multiple regression models of spherical equivalent and axial elongation after treatment.

​	Reduction of myopia progression (D) after 1-year treatment				Reduction of axial elongation (mm) after 1-year treatment			
	Beta (β)	Standard error	95% CI, %	P values	Beta (β)	Standard error	95% CI	P values
Age (years)	−0.05	0.07	−0.2 to 0.1	0.48	0.04	0.04	−0.05 to 0.12	0.40
Age at myopia onset (years)	0.09	0.07	−0.05 to 0.24	0.21	0.00	0.05	−0.1 to 0.1	1.00
Near work (hours per day)	−0.01	0.04	−0.09 to 0.08	0.88	0.01	0.02	−0.04 to 0.05	0.81
Time outdoors (hours per day)	0.15	0.11	−0.06 to 0.36	0.16	−0.05	0.06	−0.18 to 0.07	0.40
Axial length (mm)	0.04	0.11	−0.19 to 0.26	0.75	−0.06	0.06	−0.18 to 0.06	0.29
Cycloplegic spherical equivalent (D)	−0.05	0.08	−0.21 to 0.12	0.56	−0.05	0.05	−0.16 to 0.06	0.34
Sex (male as reference)	0.06	0.16	−0.26 to 0.39	0.70	−0.08	0.08	−0.25 to 0.09	0.33
Myopia progression before treatment (D)	−0.50	0.18	−0.86 to −0.14	0.01*	−0.06	0.09	−0.25 to 0.13	0.53

D, diopter. Model diagnostics were conducted: standardized residuals followed an approximate normal distribution; residual vs. fitted plots confirmed homoscedasticity. Adjusted R^2^ of the final model was 0.184 and −0.065 respectively.

* Significance was set at 0.05.

## 4 Discussion

Our study found that although there was a significant difference in myopia progression among the three groups before the treatment, the difference diminished after 1 year with 0.01% atropine eye drops. Our study showed that faster SEP before treatment is associated with a better treatment response to 0.01% atropine eye drops, with greater reduction in SEP after treatment. Children with slow SEP cannot get significant myopia control benefit from 1-year 0.01% atropine treatment. The multivariable analysis showed that the faster the myopia progression before treatment, the more significant the reduction effect of the myopia progression.

Previous studies showed children with poorer response to 1% atropine eye drops (progressed by more than −0.5 D/year) tended to be younger, to have higher myopic spherical equivalent at baseline, and to have two myopic parents (Loh et al., 2015). In another study, initial spherical refraction with less myopia was significantly associated with less myopia progression during 0.05%–0.1% atropine treatment regimen (Wu et al., 2011). However, these studies did not explore whether myopia progression in the previous, untreated year affected future myopia progression during atropine treatment. Our study showed that the faster the SEP before treatment, the more significant the reduction effect of the SEP brought by 0.01% atropine eye drops. The finding was consistent with the reports from Chia et al. demonstrating that 0.01% atropine can be an effective first-line treatment for children with the myopic progression of ≤ −0.5 D in the preceding year. They suggested a pharmacologic effect of atropine on the actively growing myopic eye (Chia et al., 2016). It also was indicated that subjects with greater myopia progression had higher treatment compliance. In the Low-Concentration Atropine for Myopia Progression (LAMP) Study, Yam et al. found that subjects with greater myopia progression in the washout phase were more likely to be re-treated with 0.05% atropine eye drops in phase 3 (Yam et al., 2019).

We further elaborate the clinical relevance of the between-group differences in myopia progression reduction observed in this study. Children of fast group obtained clinically meaningful suppression of refractive progression after receiving 0.01% atropine eye drops for 1 year. Timely intervention with 0.01% atropine eye drops for this population may effectively slow cumulative SEP throughout childhood, lowering the lifelong risk of severe pathological myopia and its associated ocular complications. Notably, children in the slow progression group showed no significant slowing of SEP after 1 year of 0.01% atropine eye drops. In fact, their SEP even showed a slight increase without statistical significance. Our results are consistent with a prior study demonstrating that premyopic children with rapid axial length elongation and SEP achieve better myopia control effects after intervention (Wang et al., 2023). Nevertheless, whether such children respond more favorably to 0.01% atropine eye drops remains unknown. This finding indicates that 0.01% atropine treatment for 1 year may provide limited or no myopia control benefit for children with slow pre-treatment SEP. This observation carries important clinical implications: 0.01% atropine treatment should be prioritized for children with fast SEP, whereas slow progressors may require alternative strategies, higher concentrations, or longer follow-up to demonstrate a treatment effect.

The possibility that severe baseline myopia of slow progression group limits further progression cannot be fully excluded. However, prior studies have indicated that annual myopia progression is similar among children aged 9 to 12 regardless of baseline refractive status (Wang et al., 2024), meaning baseline myopia only plays a minor role here. Children aged 6–14 are generally in a period of active myopia development, with median annual progression of 0.75 D at age 9 and 0.88 D at ages 10–11 (Wang et al., 2024). In our cohort, the fast progression group was younger, while the slow progression group was older. Additionally, previous literature has demonstrated that younger age correlates with a poorer response to 0.01% atropine treatment (Li F. F. et al., 2021). Even so, children with actively progressing myopia still benefited greatly from the treatment; children with naturally stable refractive development had limited efficacy of 0.01% atropine treatment.

Our study strengthens the case for personalized myopia management strategies based on individual progression profiles rather than categorical response classifications. This aligns with the perspective presented in the recent reanalysis of the LAMP study findings by Brennan et al. (Brennan et al., 2024). They contend that since an individual’s untreated progression rate is unknown, it is impossible to assess their specific treatment efficacy. Consequently, we use the absolute reduction in progression (comparing progression before and after treatment) could provide a meaningful measure of the intervention’s effect.

However, some studies’ conclusions were inconsistent with our results. In the multicenter Spanish study of 0.01% atropine treatment, Pérez et al. reported that the progression before treatment was not significantly associated with the progression during the treatment of 0.01% atropine eye drops, while researchers also noted that the problem of accurate refraction and data documentation in the past limited the retrospective comparison (Pérez-Flores et al., 2021). The confounding factors such as ethnicity, age and degree of baseline myopia may cause this discrepancy. Environmental factors such as differences in near work and outdoor time among different countries may modify the efficacy of atropine. Jeon and colleagues found that the 6-month myopia progression rate in the poor response group before treatment was higher (Jeon et al., 2021). While they reported one of the limitations of their study was that the initial myopia progression rate was not randomized, controlled, or accurate, we speculate that this may be the reason for the inconsistency with our conclusion. Also, the sample characteristics, the possible higher sensitivity to cycloplegic agents in the less pigmented Caucasian iris, and the socio-cultural and environmental differences between patients of different countries may result in the distinction among studies.

Discrepancies in the associations between risk factors and treatment responses to low-dose atropine have been reported in other studies. Zhang et al. reported a linear correlation between SE and treatment response, which showed that the risk of myopia progression declined by 14% when the baseline SE increased by 1D (Zhang et al., 2020). Other studies also reported that children with poor treatment responses had higher baseline myopia (Wu et al., 2011; Loh et al., 2015; Lee et al., 2022). Younger age was reported to be a risk factor for poor response in the LAMP study and ATOM one study, indicating that younger children require a higher concentration of atropine (Loh et al., 2015; Li F. F. et al., 2021). While our study did not find a significant relationship between age and response, neither did another long-term study of lower atropine conducted in Taiwan (Wu et al., 2011). To better explore the influence of different myopia progression rates on the atropine treatment effect, it seems necessary to set the refractive development archives to record the ocular growth in children with the plan (Chu and Qu, 2009).

The strength of the present study was that it was based on a randomized, double-masked trial. During the 2-year trial, we accurately recorded the myopia progression the year before treatment, which made us study how the rate of myopia progression before treatment affects the efficacy of 0.01% atropine eye drops. The present study has some limitations. First, the study is a *post hoc* subgroup analysis stratified by baseline myopia progression, so it is vulnerable to regression-to-the-mean bias. Given the small sample size of each subgroup, formal sensitivity analyses could not be conducted. To minimize this statistical bias, we adjusted for multiple confounders including age, baseline spherical equivalent, axial length, near work and outdoor activity in multivariable regression. Future large-sample prospective studies are required to confirm our conclusions. Second, the 38.3% dropout rate indeed reflects challenges during COVID-19 lockdowns, where clinic visits were frequently disrupted. While there was no statistical difference between lost-to-follow and continuing participants (Wei et al., 2022). Third, the pandemic-related factors (e.g., increased home-based near work, reduced outdoor time) may have confounded the myopia progression results. Finally, fast progressors were significantly younger than slow progressors. Although age was adjusted for in ANCOVA and multivariable regression models, residual confounding could not be fully excluded. In addition, participants were stratified according to baseline myopia progression, which renders the analysis vulnerable to regression-to-the-mean bias. This statistical artefact represents an alternative explanation for the larger treatment benefit observed among fast progressors. Owing to the relatively small sample size within each subgroup, formal sensitivity analyses were not feasible. Our findings should therefore be interpreted prudently. Negative results may reflect limited statistical power rather than true absence of effect, necessitating verification through adequately powered studies.

## 5 Conclusion

Faster baseline SEP before treatment is associated with a better treatment response to 0.01% atropine eye drops, with greater reduction in myopia progression after treatment. However, no significant association was found between baseline progression and axial elongation changes. Children with slow SEP cannot get significant myopia control benefit from 1-year 0.01% atropine treatment. More extensive studies with different atropine concentrations and longer follow-ups may help validate how the rate of myopia progression affects the response to 0.01% atropine eye drops.

## Data Availability

The raw data supporting the conclusions of this article will be made available by the authors, without undue reservation.
