# Supplementary material for: Pretreatment myopia progression is associated with the response to 0.01% atropine: a post-hoc secondary analysis of a randomized controlled trial
Source: Front Pharmacol. 2026 Aug 26;17:1871905. doi: 10.3389/fphar.2026.1871905 (PMC13555208; doi:10.3389/fphar.2026.1871905)
Supplement: Supplementary file 1 [file Table1.docx]

Table S1 Demographics and baseline characteristics of those completing the 2 years of follow-up

versus those who have not completed 2-year follow-up

|  | Group, mean (SD) | | |
| --- | --- | --- | --- |
|  | Completed 2 years (n = 68) | Not completed 2 years (n = 42) | *P* value |
| Age (yrs) | 10.12 (1.51) | 9.23 (1.52) | 0.02 |
| Sex (Male, %) | 34, 50.0% | 27, 64.3% | 0.17 |
| Intraocular pressure (mmHg) | 15.85 (2.49) | 15.43 (2.87) | 0.53 |
| Cycloplegic spherical equivalent (D) | -2.67 (1.42) | -2.61 (1.56) | 0.88 |
| Axial length (mm) | 24.74 (0.98) | 24.33 (0.99) | 0.1 |
| Age at myopia onset (yrs) | 8.41 (1.5) | 7.5 (1.79) | 0.03 |
| Near work (hours per day) | 3.5 (1.58) | 3.07 (1.08) | 0.23 |
| Time outdoors (hours per day) | 1.59 (0.6) | 1.5 (0.54) | 0.55 |

Abbreviation: D, diopter.
